# Long Life but High Suicide Rates in Sardinia’s Blue Zone: Evidence That Major Depression Alone May Not Explain the Paradox

**DOI:** 10.3390/jcm15218563

**Published:** 2026-11-03

**Authors:** Mauro Giovanni Carta, Massimo Tusconi, Elisa Cantone, Viviana Forte, Giulia Cossu, Michela Atzeni, Alessandra Scano, Germano Orrù, Michele Fornaro, Alessandra Perra

**Affiliations:** 1 Department of Medical Sciences and Public Health, University of Cagliari, 09124 Cagliari, Italy; 2 Department of Philosophy, Universidad del Museo Social Argentino, Buenos Aires 1723, Argentina; 3 Center for Liaison Psychiatry and Psychosomatics, University Hospital of Cagliari, 09124 Cagliari, Italy; 4 Department of Medicine and Surgery, University of Kore, Piazza dell’Università, 94100 Enna, Italy; 5 Department of Clinical Sciences, University of Cagliari, 09124 Cagliari, Italy; 6 Unit of Treatment-Resistant Psychosis, Section of Psychiatry, Department of Neuroscience, Reproductive Science and Dentistry, University School of Medicine of Naples Federico II, 80131 Naples, Italy

**Keywords:** blue zone, suicide, ageing, depression, subthreshold depression, mood dysregulation, biological rhythms, Sardinia

## Abstract

**Background**: The Sardinian Blue Zone is internationally recognized for exceptional longevity, particularly among men. The provinces encompassing this area also report elevated suicide mortality among older men. This paradox raises the question of whether older men recruited within the longevity area show increased depressive screening positivity or whether additional interacting mechanisms may contribute to suicide vulnerability. **Objective**: To examine whether the reported excess suicide mortality among older men in the provinces encompassing the Sardinian Blue Zone is accompanied by higher depressive screening positivity or PHQ-9 Item 9 scores in older men recruited within the longevity area, and to explore the distribution of subthreshold depressive symptoms. **Methods**: We conducted an exploratory secondary analysis of a population-based survey including Sardinian residents aged ≥60 years. Depressive symptoms were assessed using the Patient Health Questionnaire-9 (PHQ-9). The Blue Zone group was recruited from six municipalities within the longevity area in Nuoro and Ogliastra. Comparisons were stratified by sex and used two-sided Fisher exact tests and analysis of variance. **Results**: No statistically significant increase in screening positivity for probable depression or PHQ-9 Item 9 scores was observed in Blue Zone residents. Older men showed a higher prevalence of clinically relevant symptoms below the screening threshold for probable major depression (PHQ-9 5–9: 4/19 vs. 4/171; OR = 11.13; Fisher *p* = 0.00395). Among women, this symptom range was less frequent in the Blue Zone (5/34 vs. 77/208; Fisher *p* = 0.01077). **Conclusions**: The observed symptom distributions motivate examination of mechanisms extending beyond the prevalence of major depression alone. The male excess in subthreshold depressive symptoms, interpreted alongside independent evidence on activation, supports investigation of mood and rhythm dysregulation and culturally shaped responses to stress. The sociobiological framework generates testable hypotheses for longitudinal psychiatric and anthropological research.

## 1. Introduction

The mountainous inland region of Sardinia, particularly the provinces of Nuoro and Ogliastra, is internationally recognized as one of the world’s five Blue Zones because of its exceptional longevity and unusually high concentration of centenarians [1,2,3]. Studies conducted over the past two decades have attributed this phenomenon to a combination of a healthy lifestyle, Mediterranean dietary habits [4,5,6,7,8,9,10,11], lifelong physical activity [12,13,14], strong social cohesion [15], and genetic and epigenetic factors [16,17,18,19,20]. Together, these characteristics have made the Sardinian Blue Zone an internationally recognized model of healthy ageing [1,2,3].

A distinctive feature of the Sardinian longevity area is exceptional male longevity, with a smaller female predominance among centenarians than in many other populations [1]. This makes the reported excess suicide mortality among older men in the provinces encompassing the area particularly relevant. These population observations concern different outcomes and do not imply that the individuals contributing to exceptional longevity are those who die by suicide.

Paradoxically, however, the same geographical area has long exhibited the highest suicide rates in Italy, particularly among older men [21,22,23,24]. Epidemiological studies have consistently shown that the provinces encompassing the Blue Zone report suicide rates well above both the regional and national averages, despite their reputation for exceptional well-being in later life [21,25,26,27]. This coexistence of extraordinary longevity and elevated suicide mortality represents an unresolved epidemiological paradox. According to official regional statistics for 2006–2010, the province of Ogliastra recorded particularly elevated rates in 2008 and 2009 (17.24 and 20.66 per 100,000 inhabitants, respectively), compared with Sardinian rates of 8.95 and 8.98 and national rates of 4.74 and 4.97 in the corresponding years [28].

The mortality evidence cited here concerns the provinces encompassing the longevity area; these rates should be distinguished from rates calculated specifically for Blue Zone municipalities. Likewise, a Sardinian regional estimate is not an estimate for the complementary territory outside the Blue Zone. The survey contains no linked suicide deaths and therefore cannot describe the ages or methods of suicide among people who died in the selected municipalities.

Major depressive disorder is widely regarded as one of the strongest clinical predictors of suicide [28], and suicidal ideation is among the most robust indicators of future suicidal behavior [29,30]. Accordingly, populations with exceptionally high suicide mortality would generally be expected to exhibit a correspondingly higher prevalence of depressive disorders or suicidal ideation. Surprisingly, despite extensive research on the determinants of longevity in the Sardinian Blue Zone, this assumption has never been directly examined in older community-dwelling residents.

The present study addresses this question through a secondary analysis of a population-based survey conducted in Sardinia [31]. Specifically, we compared depressive screening positivity, subthreshold depressive symptoms, and thoughts of death or self-harm among older men living in the Blue Zone and in the remaining Sardinian provinces. Analyses among women provide complementary comparisons of the geographical symptom distribution by sex.

We hypothesized that, if an increased prevalence of major depression contributes substantially to the reported excess suicide mortality, older men in the Blue Zone would show more frequent depressive screening positivity and higher PHQ-9 Item 9 scores than older men elsewhere in Sardinia. An absence of statistically significant differences would not exclude such a contribution, but a distinct subthreshold symptom pattern could motivate investigation of additional interacting pathways. These may involve depressive presentations, mood and energy regulation, biological and social rhythms, and culturally shaped responses to stress.

## 2. Materials and Methods

### 2.1. Study Design and Participants

This study is a secondary analysis of data derived from a previously published population-based survey conducted in Sardinia, Italy, to investigate mental health and public attitudes toward depression [31].

The original survey included community-dwelling individuals aged 16 years or older and was carried out using a Computer-Assisted Telephone Interviewing (CATI) system. A stratified sampling design was adopted to obtain a representative sample of the Sardinian population. The island was divided into its eight administrative provinces, each further stratified into the provincial capital and the remaining municipalities. Within each stratum, participants were selected according to age and sex using demographic data provided by the Italian National Institute of Statistics (ISTAT) [32]. Interviews were conducted by trained physicians and psychologists experienced in mental health assessment [31].

The present secondary analysis was restricted to participants aged 60 years or older, as suicide mortality in Sardinia is concentrated predominantly among older adults.

### 2.2. Study Groups

Provincial labels identify the administrative location of recruitment, rather than implying that all residents of Nuoro and Ogliastra belong to the Blue Zone. Municipality information was retained in the original dataset. All Ogliastra participants were selected in three municipalities within the Sardinian Blue Zone; Nuoro participants were selected in two municipalities within the zone and one larger municipality at its boundary, but within the perimeter adopted for this study. The geographical framework follows the longevity area described in the AKEA research [1,2]. Municipality names are withheld to protect confidentiality in small geographical and demographic subgroups. Residents of the remaining Sardinian provinces constituted the comparison group (Other Sardinian Provinces). Published provincial suicide mortality provides the epidemiological context and is distinct from the municipality-based recruitment classification.

### 2.3. Measures

Demographic variables included age, sex, and province of residence.

Depressive symptoms were assessed using the Patient Health Questionnaire-9 (PHQ-9) [33,34,35], administered in its validated Italian version [34,36]. The PHQ-9 is a widely used self-report instrument that evaluates the nine DSM-defined symptoms of major depressive disorder over the preceding two weeks, with each item scored from 0 (“not at all”) to 3 (“nearly every day”) [37].

Participants with a PHQ-9 total score ≥10, equivalent to >9, were considered screen-positive for probable depression according to the conventional screening threshold [33,38]. No diagnostic interview established major depressive disorder or a major depressive episode in this secondary analysis; screening positivity is therefore distinguished from a confirmed clinical diagnosis.

The PHQ-9 has also been evaluated in older populations, including primary-care patients aged ≥65 [39], a community sample of Korean adults aged >60 [40], and hospitalized Polish patients aged ≥60 [41]. These studies support its relevance to later-life depressive symptom assessment, while their differing optimal thresholds emphasize that screening performance depends on setting and population.

PHQ-9 scores of 5–9 were classified as clinically relevant mild depressive symptoms below the conventional screening threshold for probable major depression. This interval corresponds to the mild severity category in the original validation study [33]; it is not a separate diagnostic entity, and scores below 10 do not individually rule out a major depressive episode [38,42].

The decision to examine this interval separately was informed by Urban et al. [43], who investigated thoughts of death or self-harm below the PHQ-9 threshold for probable major depression and assessed accompanying energy and rhythm profiles using SF-12 Item 10 and BRIAN. The small subgroup described in that study showed a profile compatible with DYMERS on direct assessments of energy and rhythm regulation. It therefore provides partial empirical support for the clinical relevance of these subthreshold presentations, in addition to the rationale for investigating them. The present analysis focuses on the conventional 5–9 symptom range; this grouping does not itself identify DYMERS, hyperenergy, hyperarousal, mood instability, or rhythm dysregulation.

Thoughts of death or self-harm were assessed using PHQ-9 Item 9 (“Thoughts that you would be better off dead or of hurting yourself in some way”) over the preceding two weeks. The item includes passive death wishes and thoughts of self-harm, but does not distinguish them or assess suicidal planning, intent, access to means, or previous behaviour. Large longitudinal studies demonstrate associations between Item 9 responses and subsequent suicide attempts and deaths, including in older adults [30,44,45,46]. Depressive symptom severity assessed through the other PHQ items is also associated with subsequent suicide mortality [46]. These findings establish the relevance of the survey measures to suicide epidemiology, although their prognostic performance in clinical cohorts cannot be assumed to transfer quantitatively to this community sample. Item 9 scores were compared as mean scores between groups.

### 2.4. Statistical Analysis

The primary hypothesis was that, if an increased prevalence of major depression contributes substantially to the reported excess suicide mortality, older men living in the Sardinian Blue Zone would exhibit more frequent screening positivity for probable depression and higher PHQ-9 Item 9 scores than older men living in the remaining Sardinian provinces. Comparisons among women were complementary.

The primary outcome was screening positivity for probable depression (PHQ-9 ≥10).

Secondary outcomes included the prevalence of clinically relevant depressive symptoms below the screening threshold for probable major depression (PHQ-9 score 5–9), total PHQ-9 scores, and PHQ-9 Item 9 scores.

Because reported excess suicide mortality predominantly affects older men, the principal comparisons were stratified by sex. Pooled estimates were additionally standardized directly to the sex distribution of the full analytical sample (242/432 women and 190/432 men), using a common reference composition rather than survey weights. No survey sampling weights were applied.

Binary categorical comparisons used two-sided Fisher exact tests because several cells were sparse. Sample odds ratios (ORs) were reported with exact conditional 95% confidence intervals (CIs); for zero-event cells, the exact interval was retained even where the sample OR was zero or infinite. Continuous outcomes were compared using one-way analysis of variance (ANOVA). Statistical significance was defined as *p* < 0.05. These exploratory comparisons were not adjusted for multiplicity; interpretation considers both the number of comparisons and estimate precision. A sensitivity analysis calculated the power of the two-sided Fisher test at α = 0.05 for the male sample sizes (19 and 171), summing rejection probabilities under independent binomial distributions across specified alternative prevalences. This analysis describes detectable contrasts, rather than calculating observed power from the estimated effect [47]. As a complementary analysis, PHQ-9 Item 9 responses were dichotomized as 0 versus ≥1 and compared between geographical groups using two-sided Fisher exact tests.

Complete PHQ-9 item-level data were available for all 432 participants included in the analysis; no imputation was required.

### 2.5. Ethical Considerations

The original survey was approved by the Ethics Committee of the University Hospital of Cagliari (Protocol No. 139, 14 July 2011). All participants provided informed consent before participation, and the study was conducted in accordance with the principles of the Declaration of Helsinki.

## 3. Results

### 3.1. Sample Characteristics

A total of 432 participants aged 60 years or older were included in the descriptive analysis, of whom 53 were classified in the Blue Zone group (Nuoro and Ogliastra) and 379 in the remaining Sardinian provinces (Table 1).

Women represented 64.1% of the Blue Zone group and 54.9% of the comparison group. Mean age was similar among women across areas. Men in the Blue Zone were on average 3.36 years younger than men in the comparison group, although the difference was not statistically significant (Table 1). These descriptive comparisons do not exclude confounding by age.

### 3.2. Screening Positivity for Probable Depression

The prevalence of screening positivity for probable depression did not differ significantly between residents of the Blue Zone and those living in the remaining Sardinian provinces (Table 2).

Among women, screening positivity was 5.9% in the Blue Zone and 13.9% elsewhere (Fisher *p* = 0.271). Among men, no Blue Zone participant screened positive, compared with 5.8% elsewhere (Fisher *p* = 0.602). The exact two-sided 95% binomial CI for 0/19 was 0–17.6%, illustrating the uncertainty attached to this zero-event estimate.

No increase in screening positivity was detected in this sample. Sex-standardized prevalence was 3.3% in the Blue Zone and 10.4% in the other provinces; these pooled descriptive estimates do not establish equivalence between populations.

### 3.3. Clinically Relevant Depressive Symptoms Below the Screening Threshold

When participants with clinically relevant depressive symptoms below the screening threshold for probable major depression (PHQ-9 score 5–9) were examined separately (Table 3), a different pattern emerged.

Among women, symptoms in this range were less frequent in the Blue Zone than elsewhere (14.7% vs. 37.0%; Fisher *p* = 0.01077).

Among men, symptoms in this range were substantially more frequent in the Blue Zone (21.1% vs. 2.3%; sample OR = 11.13; exact conditional 95% CI reported in Table 3; Fisher *p* = 0.00395). The estimate is based on four cases in each group.

For male samples of 19 and 171, exact Fisher-test power at α = 0.05 was 10.7%, 28.3%, and 49.2% for specified Blue Zone prevalences of 10%, 15%, and 20%, respectively, against 6% elsewhere. These hypothetical alternatives were selected to assess sensitivity for moderate-to-large differences in probable depressive episode prevalence; they are not estimates of the true prevalences.

The pooled comparison was not statistically significant (Fisher *p* = 0.588); sex-standardized prevalence was 17.5% in the Blue Zone and 21.8% elsewhere. The opposite directions of the sex-stratified contrasts are therefore more informative than the pooled estimate.

### 3.4. Thoughts of Death or Self-Harm

Mean scores on PHQ-9 Item 9, assessing thoughts of death or self-harm, did not differ significantly between residents of the Blue Zone and those living in the remaining Sardinian provinces (Table 4).

Within-area comparisons of Item 9 scores between men and women were also nonsignificant (Table 4). These comparisons are not formal tests of a sex-by-area interaction.

No statistically significant increase in Item 9 scores was detected in the Blue Zone sample. This comparison concerns the frequency of thoughts captured by a two-week screening item, rather than a comprehensive assessment of suicidality.

As a complementary analysis, PHQ-9 Item 9 responses were dichotomized as 0 versus ≥1 to describe the proportion of participants reporting any thoughts of death or self-harm during the preceding two weeks. Positive responses were reported by 2/34 women (5.9%) in the Blue Zone and 18/208 women (8.7%) in the other Sardinian provinces (two-sided Fisher exact *p* = 0.748). Among men, the corresponding proportions were 1/19 (5.3%) and 9/171 (5.3%), respectively (*p* = 1.000). Overall, positive responses were reported by 3/53 Blue Zone participants (5.7%) and 27/379 participants elsewhere (7.1%; *p* = 1.000).

## 4. Discussion

### 4.1. Main Findings

The present study sought to determine whether the elevated suicide mortality reported among older men in the provinces encompassing the Sardinian Blue Zone is accompanied by higher depressive screening positivity and PHQ-9 Item 9 scores among older men recruited within the longevity area.

No statistically significant increase in screening positivity for probable depression or thoughts of death or self-harm was detected in Blue Zone residents. These are relevant epidemiological observations because depression and PHQ-based indicators have documented associations with subsequent suicidal outcomes [44,45,46,48,49]. The study examines their distribution in a setting of reported excess suicide mortality, rather than directly following individuals for suicidal outcomes.

An exploratory sex-stratified contrast was observed below the PHQ-9 screening threshold for probable major depression. Among men, PHQ-9 scores of 5–9 occurred in 4/19 Blue Zone participants versus 4/171 men elsewhere (OR = 11.13; exact 95% CI, 1.83–64.69). Among women, the corresponding proportion was lower in the Blue Zone than elsewhere. Given the sparse male events, wide confidence interval, and multiple unadjusted comparisons, these findings should be considered hypothesis-generating rather than confirmatory; no formal sex-by-area interaction was tested.

### 4.2. Depression Alone May Not Explain the Blue Zone Paradox

The coexistence of exceptional longevity and persistently high suicide mortality among older men represents one of the most intriguing epidemiological paradoxes in European mental health.

Our starting hypothesis was that excess suicide mortality driven primarily by an increased prevalence of major depression would be accompanied by more frequent depressive screening positivity and thoughts of death or self-harm. The observed distributions did not show that pattern. Although limited precision precludes ruling out an increase, the male excess in the subthreshold range provides a positive finding that motivates examination of additional pathways.

The findings do not exclude an important contribution of depression or other psychopathological processes to the mortality pattern. The male excess in the mild symptom range warrants further characterization alongside the absence of a detected increase in PHQ-9 screening positivity. The proposed framework therefore examines interactions between depressive presentations, mood and energy regulation, biological and social rhythms, and culturally shaped responses to threats to autonomy or social standing. This formulation is consistent with suicide vulnerability as the product of interacting biological, psychological, and sociocultural processes [50,51,52].

### 4.3. A Possible Role for Subthreshold Mood Dysregulation

The observed contrast in mild depressive symptoms (PHQ-9 scores of 5–9) among older Blue Zone men is based on only four cases in each geographical group and must be interpreted as exploratory and hypothesis-generating, not as confirmation of a distinct clinical phenotype. Importantly, the PHQ-9 is a validated measure of depressive symptom severity and does not assess mood instability, hyperenergy, hyperarousal, or biological and social rhythm dysregulation. Accordingly, scores within this range should not be interpreted as diagnostic or surrogate indicators of Dysregulation of Mood, Energy, and Social Rhythms Syndrome (DYMERS). Nevertheless, the clinical relevance of investigating subthreshold depressive presentations in relation to these additional dimensions is supported by preliminary evidence from Urban et al. [43]. In their study of healthcare workers, individuals reporting death-related or self-harm thoughts despite not meeting the PHQ-9 screening threshold for probable major depression exhibited, on descriptive assessment, a profile suggestive of increased activation and rhythm dysregulation, evaluated independently using SF-12 Item 10 and the Biological Rhythms Interview of Assessment in Neuropsychiatry (BRIAN) [53,54,55], respectively. Although these observations were based on a very small subgroup and cannot establish a specific syndrome or a causal association, they provide an empirical rationale for investigating whether depressive symptoms below the conventional screening threshold may coexist with disturbances in energy and rhythm regulation. In the present study, such dimensions were not directly measured; therefore, any potential relationship between the observed PHQ-9 symptom distribution and DYMERS-related characteristics remains strictly hypothetical and requires confirmation through dedicated clinical assessments and longitudinal investigations.

Independent observations from the same geographical setting strengthen this question. In a separate clinic-recruited sample, Scano et al. found MDQ positivity in 22.58% of the oldest Blue Zone participants without bipolar disorder, alongside preserved quality of life and no corresponding increase in depressive symptoms or rhythm dysregulation [56,57]. Here, hyperenergy refers to increased energy and activity considered within an activation profile, rather than a diagnosis of hypomania; in that study, the profile was assessed using MDQ and clinical evaluation. These individuals were not participants in the present survey. Together, the studies motivate investigation of whether an ordinarily adaptive activation profile may shape responses to stress and, in some individuals, become associated with subthreshold distress.

Recurrent Brief Depression has been associated with substantial suicidal risk despite the short duration of depressive episodes [58,59,60,61]. DYMERS was introduced as a heuristic transdiagnostic construct encompassing dysregulation of mood, energy, and social rhythms [62], with subsequent research examining rhythm disturbances and impaired quality of life among MDQ-positive people [63]. These frameworks are relevant to presentations extending beyond categorical major depression and to sex distributions that may differ from its usual female predominance. The present findings motivate targeted assessment of these profiles, including their possible role in the transition from distress or suicidal thoughts to suicidal behaviour.

Cyclothymic temperament provides a complementary framework for investigating fluctuations in mood, energy and activity. In a clinical sample of 2561 patients with mood or anxiety disorders, cyclothymic and dysthymic temperaments were associated with suicidal ideation and acts, whereas hyperthymic temperament showed an inverse association [64]. These observations support targeted investigation of affective temperament, while its distribution and contribution to mortality among older Blue Zone residents remain to be assessed. Cyclothymic temperament, clinically diagnosed cyclothymic disorder, DYMERS-related dysregulation and adaptive high energy are distinguishable constructs. Their possible overlap can be examined using dedicated temperament measures such as TEMPS-A alongside longitudinal clinical assessment and direct energy and rhythm measures.

### 4.4. Theoretical Considerations

The proposed sociocultural framework concerns interactions with psychopathological processes rather than assuming that psychopathology has a secondary role. The survey does not comprehensively assess psychopathology and cannot establish its overall contribution to the mortality pattern.

Sardinian anthropological accounts describe masculine honour, responsibility, and social reputation within pastoral communities, including the concept of balentia [65,66,67,68]. Historical research on suicide and homicide in small Sardinian communities also directs attention to social cohesion and normative expectations [26]. These sources provide a cultural basis for considering how threats to autonomy, social role, or reputation may shape responses to distress.

Durkheim’s concept of altruistic suicide [69] offers an interpretive framework for settings where obligations to family and community are especially strong. Within this framework, perceived loss of autonomy or social standing may have particular significance for older men. This is an anthropological interpretation to be investigated, rather than a classification of the individual suicides or participants considered here.

The cultural account can be integrated with a hypothesis about adaptation to longstanding ecological and social demands. Pastoral work involving transhumance, mobility, environmental uncertainty, and autonomous decisions [70] may have favoured exploratory behaviour, sustained activation, and distinctive stress responses. These dispositions could be adaptive under ordinary conditions yet contribute to dysregulated responses when social or economic threats become overwhelming.

An anthropological hypothesis of historical adaptation can arise from ethnography, historical reconstruction, and comparisons across ecological settings; it need not originate from genetic evidence. Historical processes cannot be experimentally reproduced, but their proposed consequences generate testable predictions. Comparative studies and natural experiments offer methods for examining such questions [71], and cultural phylogenetic approaches can assess biocultural hypotheses while accounting for shared ancestry [72]. Cultural transmission, developmental adaptation, and genetic selection are distinguishable mechanisms; evidence for contemporary associations would not by itself establish historical genetic selection.

The proposed sociobiological explanation should be regarded as a speculative theoretical hypothesis rather than an empirically established mechanism. Neither the cultural exposures nor the DYMERS-related dimensions proposed to interact with them were directly assessed in the present study. Nevertheless, this framework generates testable hypotheses concerning potential interactions between pastoral background, masculine role expectations, energy and rhythm regulation, and responses to threats to autonomy or social standing. These hypotheses require investigation in larger, adequately powered comparative and longitudinal studies incorporating direct clinical, psychometric, and sociocultural assessments.

### 4.5. Implications and Future Directions

The exploratory findings generate hypotheses for future research rather than establishing a distinct clinical phenotype or a mechanism underlying suicide mortality in the Sardinian Blue Zone. Larger, adequately powered studies are needed to determine whether the observed subgroup differences can be replicated and whether directly measured mood, energy, and rhythm dysregulation, together with sociocultural factors, contribute to suicide vulnerability.

Future studies should combine clinical assessments of depressive episodes, recurrent brief presentations, mood instability, energy, hyperarousal, and biological and social rhythms with anthropological measures of role expectations and stress responses. Linkage to regional mortality registries and records of suicide attempts and self-harm would establish whether these profiles predict individual outcomes and help explain the geographical excess in suicide mortality.

A combined assessment using the PHQ-9, geriatric-specific instruments such as the Geriatric Depression Scale (GDS), and clinical evaluation could broaden the characterization of later-life depressive symptoms. PHQ-9 Item 6 addresses negative self-evaluation and feelings of failure, while Item 2 includes hopelessness. However, these experiences are not separately characterized in depth, and helplessness, life satisfaction and perceived loss of social usefulness are not specifically assessed. These dimensions may help clarify relationships between depressive symptoms, autonomy, social value and balentia. The GDS was not administered in the original survey and cannot be added retrospectively; complementary instruments would extend characterization rather than invalidate the standardized PHQ-9 comparisons, which have supporting evidence in older adults [33,58,59,60].

## 5. Limitations

The principal limitations concern precision and the scope of mechanistic inference.

The original survey assessed mental health and public attitudes toward depression rather than the full range of suicide determinants. The present analysis lacked comprehensive information on psychiatric history, previous suicidal behaviour, medical comorbidity, social isolation, economic stressors, and impulsivity. This restricts examination of alternative explanations and interactions, while the PHQ-9 measures retain their established relevance to suicidal outcomes [44,45,46].

No suicide deaths, attempts, or suicide-related hospitalizations were linked to participants. Consequently, the study compares established suicide-associated indicators across geographical groups but cannot estimate their individual contribution to mortality or demonstrate causal mediation. Published provincial mortality and the municipality-selected survey also describe different geographical scales. The inclusion of a municipality at the boundary of the Blue Zone may introduce some geographical classification uncertainty, as the included localities may not share identical longevity characteristics.

PHQ-9 scores of 5–9 indicate mild depressive symptom severity rather than a distinct clinical syndrome, and cannot independently identify DYMERS, mood instability, hyperenergy, hyperarousal, or disturbances in biological and social rhythms. Although previous exploratory research has investigated the coexistence of subthreshold depressive symptoms with activation and rhythm abnormalities using dedicated instruments [43], these dimensions were not assessed in the present dataset. Consequently, the proposed DYMERS-related interpretation represents a hypothesis derived from external evidence rather than a finding directly supported by our measurements. Furthermore, PHQ-9 Item 9 captures death-related or self-harm thoughts without establishing suicidal intent or planning. Future studies should incorporate direct measures of energy, affective instability, and biological rhythms, alongside structured clinical evaluations, to determine whether these dimensions characterize specific subgroups of older adults presenting with mild depressive symptoms.

The subgroup of 19 Blue Zone men yields imprecise estimates, particularly for sparse or zero-event outcomes. Nonsignificant comparisons do not demonstrate equivalence. The sensitivity analysis illustrates limited power for moderate prevalence differences, and multiple exploratory comparisons create a risk of chance findings. Exact inference improves handling of sparse cells but does not remove these limitations. Specifically, the 4/19 versus 4/171 male contrast is based on only eight events, and its wide exact confidence interval limits the stability and generalizability of the estimated association; independent replication in larger, adequately powered samples is needed.

Balentia, pastoral role expectations, and adaptive activation were not measured in this survey. Their integration rests on anthropological evidence and independent clinical studies. The model’s value lies in the explicit predictions it generates for comparative, longitudinal, and suitably designed quasi-experimental research.

Within this scope, the study contributes a distinctive sex-related symptom pattern and a focused framework for examining suicide vulnerability beyond the prevalence of major depression alone.

## 6. Conclusions

The Sardinian Blue Zone represents a unique natural model in which exceptional longevity coexists with one of the highest suicide rates among older men in Italy. This apparent contradiction provides an opportunity to re-examine current models of suicide vulnerability.

No statistically significant increase in screening positivity for probable depression or PHQ-9 Item 9 scores was detected in the Blue Zone sample. A higher proportion of PHQ-9 scores of 5–9 was observed among men (4/19 vs. 4/171), but this sparse-data comparison has substantial uncertainty and should be regarded as hypothesis-generating rather than confirmatory. It does not establish that pathways beyond major depression explain the regional suicide mortality pattern.

The present survey did not assess DYMERS, hyperactivation, mood instability, or biological and social rhythm dysregulation. Therefore, the observed PHQ-9 symptom distributions provide no direct evidence for these constructs or their involvement in suicide vulnerability. Independent findings discussed above provide a theoretical rationale for investigating these dimensions in future studies, but cannot establish their presence or explanatory role in the current sample.

The proposed DYMERS-based and sociobiological explanations remain speculative theoretical hypotheses that cannot be established from the present data. Their potential relevance should be investigated in larger, adequately powered longitudinal studies incorporating direct assessments of mood, energy, biological and social rhythms, and sociocultural factors, ideally with linkage to individual suicidal outcomes. Such investigations may help determine whether these mechanisms contribute to suicide vulnerability in the Sardinian Blue Zone.

## Figures and Tables

**Table 1:** Age and sex distribution by area of residence.

Sex	Blue Zone *n*	Age Mean ± SD	Other Provinces *n*	Age Mean ± SD	*p* Value
Women	34	71.29 ± 8.18	208	71.25 ± 8.36	0.979
Men	19	67.47 ± 8.78	171	70.83 ± 8.20	0.126
Total	53	69.94 ± 8.51	379	71.06 ± 8.28	0.370

Age is expressed in years as mean ± SD. *p* values compare age between areas using two-sided Welch *t* tests.

**Table 2:** Screening positivity for probable depression (PHQ-9 ≥10): Blue Zone vs. Other Sardinian Provinces.

Residence	Women Screen-Positive for Probable Depression *n*/N (%)	Men Screen-Positive for Probable Depression *n*/N (%)	Total Screen-Positive for Probable Depression *n*/N (%)
Other Sardinian Provinces	29/208 (13.9%)	10/171 (5.8%)	39/379 (10.3%)
Blue Zone Provinces	2/34 (5.9%)	0/19 (0%)	2/53 (3.8%)
	Fisher exact test (two-sided)	Fisher exact test (two-sided)	Fisher exact test (two-sided)
	*p* = 0.27102	*p* = 0.60206	*p* = 0.20655
	OR (OSP) = 2.59 Exact 95% CI 0.60–23.43	OR (OSP) = ∞ Exact 95% CI 0.24–∞	OR (OSP) = 2.92 Exact 95% CI 0.72–25.71

Legend: OR = sample odds ratio; CI = exact conditional confidence interval; OSP = Other Sardinian Provinces. PHQ-9 ≥10 indicates screening positivity, not a confirmed diagnosis. All *p*-values are two-sided Fisher exact tests.

**Table 3:** Clinically relevant depressive symptoms below the screening threshold, PHQ-9 score 5–9 (STDS).

Residence	Women with STDS *n*/N (%)	Men with STDS *n*/N (%)	Total with STDS *n*/N (%)
Other Sardinian Provinces	77/208 (37.0%)	4/171 (2.3%)	81/379 (21.4%)
Blue Zone Provinces	5/34 (14.7%)	4/19 (21.0%)	9/53 (17.0%)
	Fisher exact test (two-sided)	Fisher exact test (two-sided)	Fisher exact test (two-sided)
	*p* = 0.01077	*p* = 0.00395	*p* = 0.58831
	OR (OSP) = 3.41 Exact 95% CI 1.23–11.70	OR (BZ) = 11.13 Exact 95% CI 1.83–64.69	OR (OSP) = 1.33 Exact 95% CI 0.61–3.23

Legend: OR = sample odds ratio; CI = exact conditional confidence interval; OSP = Other Sardinian Provinces; BZ = Blue Zone. PHQ-9 5–9 denotes subthreshold mild symptoms. All *p*-values are two-sided Fisher exact tests.

**Table 4:** PHQ-9 Item 9 (Thoughts of death/self-harm).

Residence	Item 9 Women (Mean ± SD)	Item 9 Men (Mean ± SD)	Men vs. Women
Blue Zone Provinces	0.08 ± 0.37 (N = 34)	0.10 ± 0.30 (N = 19)	F(1,51) = 0.001, *p* = 0.999
Other Sardinian Provinces	0.17 ± 0.47 (N = 208)	0.19 ± 0.47 (N = 171)	F(1,377) = 0.170, *p* = 0.680
Geographical comparison ANOVA	F (1,240) = 1.131, *p* = 0.289	F (1,188) = 0.665, *p* = 0.416	

Legend: SD = standard deviation.

## Data Availability

The datasets of this study will not be publicly available due to individual privacy rules.

## Abbreviations

The following abbreviations are used in this manuscript:

ISTAT	Italian National Institute of Statistics
CATI	Computer-Assisted Telephone Interviewing
PHQ-9	Patient Health Questionnaire Nine Item Version
ANOVA	Analysis of Variance
OR	odds ratio
CI	confidence interval
DSM	diagnostic and statistical manual of mental disorders
Df	Degrees of Freedom
MDD	Major Depressive Disorder
STDS	Sub-Thresold Depressive Symptoms
OSP	Other Sardinian Provinces
BZP	Blue Zone Provinces (Nuoro and Ogliastra)
